# Diet of moulting Swainson's Thrushes (*Catharus ustulatus*) and Tennessee Warblers (*Leiothlypis peregrina*) at a stopover site during fall migration measured with fecal DNA metabarcoding

**DOI:** 10.1038/s41598-024-59462-0

**Published:** 2024-04-30

**Authors:** Ana Blanc-Benigeri, Vanessa Poirier, Desiree Narango, Kyle H. Elliott, Barbara Frei

**Affiliations:** 1 Department of Natural Resources Sciences, McGill University, Montreal, QC Canada; 2 Vermont Center for Ecostudies, White River Junction, Hartford, VT 05001 USA; 3 Science and Technology Branch, Environment and Climate Change Canada, Montreal, Canada

**Keywords:** Animal migration, Conservation biology, Urban ecology, Animal behaviour

## Abstract

Moult and migration are energetically demanding and require adequate nutrition. In some species, individuals may interrupt their fall migration to moult at discrete stopover locations outside of their breeding grounds (i.e., moult-migration) leading to competing nutritional demands for moult and migration. Here, we use DNA barcoding of fecal samples to compare the diet of moulting and actively migrating (post-moult) Swainson’s Thrushes (*Catharus ustulatus*) and Tennessee Warblers (*Leiothlypis peregrina*) during their fall migration stopover at a large urban greenspace in Montreal, Canada. Diet differed according to moult status, species, and seasonality. Swainson’s Thrushes had a broad diet with frequent detections of both insects and berry-producing shrubs; while detections in Tennessee Warblers’ diets were mainly arthropods. For both species, more actively migrating individuals consumed fleshy-fruiting plants than moulting individuals. A higher proportion of moulting birds consumed arthropods compared to active migrants, due to either arthropod availability or a dietary preference for proteinaceous foods to grow feathers. Both species and moult classes consumed more native plants than non-native plants later in the season. We show the importance of managing urban greenspaces with native plants and diverse food sources that can provide for the different dietary needs of migratory birds.

## Introduction

The annual life cycle of a vast number of birds include migration between their breeding and wintering grounds^1^, which is an energetically demanding period, increasing a songbird’s energy expenditure by up to 30%^2^. Most north-temperate adult passerines undergo a complete prebasic moult—the loss and regrowth of all feathers—following the breeding season to prepare for their migration^3^. However, some individuals of certain migratory bird species will start their migration and halt at specific stopover sites outside of their breeding grounds to moult their worn-out feathers; they are called moult-migrants^3–7^. The various sequential stages of bird’s life cycle (breeding, moult, migration, etc.) have separate nutritional demands, with migrating bird needing to store fat reserves (fuel) and rebuild muscle mass (power) to power and fuel their long-distance flights between their breeding and wintering grounds^8,9^. Alternatively, birds undergoing post-breeding moult could face higher protein demands when replacing body and flight feathers because moult requires protein for feather growth^3,4,10^. Moulting birds may have a lower physiological capacity for building fat stores at that time^11^, which is why stopover sites have a critical role in providing migrants areas with key resources to replenish their fat and muscle mass before carrying on with their migration^5,11,12^.

Stopover sites are especially important for birds in active moult, as they have exceptionally high energetic demands during this period^11^. Individuals going though their flight feather moult are likely to have an impaired flight efficiency, which could suggest the need for specific habitat requirements at stopover sites, differing to the ones at their breeding and wintering sites^4^. For example, Tennessee Warblers (*Leiothlypis peregrina*) moult migrants in our study area have been observed to have large overlapping stopover home ranges (∼15 ha) and were dependent on high abundance of forest and forest edge^13^. Food nutritional quality and abundance are crucial for efficient refueling and can influence the time spent at stopover sites, since those affect the rate at which birds replace their energy stores^14,15^. Buler et al^12^. show that densities of insectivorous migrants are positively related to arthropod abundance while frugivorous migrants are positively related to the amount of fleshy-fruited plants during autumn^12^.

Many insectivorous bird species breeding in North America switch to a partially frugivorous diet during their fall migration^8,15^. This dietary plasticity allows birds to exploit resources that are more abundant during autumn^15^. Dietary flexibility may have evolved due to the spatial and temporal uncertainty in insect availability during autumn stopover^8,16^. Migrants eating fruits use less energy-expensive foraging techniques, encounter more food items per unit of time, and execute fewer search movements than when feeding on insects^8^. Therefore, the use of temporally and spatially stable fruit resources decreases searching and handling time, which in turn decreases energy expenditure during foraging and results in a positive net energy budget for migrants^2,8,16^. Due to seasonal frugivory, many species select different habitats than those preferred at other times of the year to optimize their energy gain^17^. Highly frugivorous migratory species can refuel at a faster rate when fruit diversity, quality, and availability are high^15^.

Evidence also suggests that migratory passerines tend to prefer native over non-native plant species for feeding. Oguchi et al. (2017) showed that fall migrating Swainson's Thrushes (*Catharus ustulatus*) and Gray Catbirds (*Dumetella carolinensis*) selected foraging habitat and occurred at higher densities in predominantly native shrublands rather than in exotic-dominated shrubland habitats^18^. Smith et al^14^. demonstrated that certain native fruiting shrub species are of higher nutritional quality to songbirds during fall migration compared to invasive species due to high lipid content. Indeed, native fruits tend to have greater fat and energy density, whereas exotic fruits tend to contain more sugar and water^18^.

These energetic differences between native and non-native fruits can have implications for the foraging efficiency for migratory species. For example, it is estimated that birds need to consume up to three times the wet mass of non-native exotic *Elaeagnus umbellata* fruits compared to the ones of native *Ilex verticillata* to obtain the same amount of energy^18^. Thus, fall migrants should generally prefer lipid- and energy-rich fruits^18^. Unfortunately, anthropogenic alterations of stopover habitats such as introduction and spread of non-native plants have been identified as a major conservation concern^18^. Invasive plant species can drastically change habitat quality due to their relatively high growth rate and competitive ability compared to native plants^14^. This can result in substantial environmental costs, particularly if the non-native plants compete with the native ones that provides important food resources for migrating birds. Additionally, non-native plants negatively impact insect availability and abundance since they are poor at supporting the insect communities that are critical food resource for birds, notably communities with high lipid and protein like caterpillars and spiders^19,20^. The protein requirements of many migratory birds can be met by eating higher-protein insects, which is why shrubland habitat can be important migratory stopover sites since they contain a variety of preferred fruit-bearing shrubs and an adequate abundance of insects^17^.

Although the current understanding of moult and plumage cycles is limited, moult-migration seems to be more common than currently recognized for passerines in North America^4,7^, so documenting it should be a priority. Additionally, existing conservation plans may lack information to fully maintain and improve stopover sites, as Neotropical migrants' habitat and food requirements during their migratory period remain insufficiently studied compared to the breeding and wintering seasons^21^. Data on the food requirements of Neotropical migrants throughout their complete annual cycle would benefit species conservation. Indeed, moult-stopovers are critical locations to conserve because moulting birds are highly vulnerable at this time^4,11^ and they stop for long periods at these sites (i.e. approximately 1–1.5 months, representing 13% of the annual cycle for Swainson’s Thrushes)^5^. Existence of diet differentiation among the migratory songbirds, specifically regarding the ecology of the moult migrants, would provide an important new insight into identifying critical moulting stopover requirements.

To better understand food items used by birds during stopover, we describe and compare the diet of two neotropical migrants and boreal forest breeders (Swainson’s Thrush and Tennessee Warbler) during their post-moult and moult migration in a peri-urban greenspaces of Montreal, Quebec, Canada. These songbird species are known to have differing diet preferences between seasons, and the two species feed at different foraging heights. Breeding and spring migrating populations of Swainson’s Thrush tend to be insectivorous whereas fall migrating and wintering populations are more frugivorous^16,22–24^. Swainson’s Thrushes are predominantly near-ground foragers (< 2 m high), whereas Tennessee Warblers are arboreal and insectivorous, but are opportunistic fruit eaters and nectar robbers during migration^25–27^. Despite the general knowledge of shifting diets based on species and seasonality, researchers have not yet identified specific diet requirements during the vulnerable period of moult migration and how this might compare to actively migrating individuals. We used fecal DNA metabarcoding to examine the composition of Swainson’s Thrushes and Tennessee Warblers' diets and to determine if diets changed according to moulting status or species. First, we predict more Swainson’s Thrush fecal samples to contain plant DNAs and more Tennesee Warbler samples to contain arthropod DNAs, as described in previous diet studies. As birds undergoing moult migration have various competing nutritional demands (i.e. protein to replace feathers and sugars to fuel for migration), we hypothesise dietary differences between moulting and post-moult migrants^8,16^. We predict moult-migrants to have a primarily insectivorous diet and post-moult migrants to have a berry-rich, frugivorous diet.

## Methods

### Study site and species

We conducted our fieldwork at the McGill Bird Observatory (MBO) near the Macdonald Campus of McGill University, on the west side of the island of Montreal (45.43°N, 73.94°W;^5,6^. The migration monitoring station is part of the “Montreal Gap”, a 600 km section of land in between Lake Ontario and the Gulf of the St Lawrence, through which migrating birds pass and avoid bodies of water when carrying out their continental migration^28^. The MBO is within the *Grand Parc de l’Ouest* nature reserve, which extends over more than 3000 ha^2^.

We surveyed the common fruit bearing species available at the MBO for fruit availability. We sampled the present shrubs—woody plants between 0.5 and 3 m tall—within 11 random 2 m-radius circular quadrats inside the study site. Quadrats were evenly distributed across the study site by randomly selecting a distance from either close (0–50 m) or far (50 m–125 m) from the banders’ cabin (i.e. where we would release the bird) and an angle (from 0 to 360 degrees) which would be the center of the quadrat. All random selections were done using a random number generator cellular application. Surveys were performed in 2021 (July to September) and 2022 in (June–July). The most common native fleshy-fruiting shrubs found were hackberry (*Celtis occidentalis*), purple-flowering raspberry (*Rubus odoratus*), dogwood (*Cornus sp.*), hawthorn (*Crataegus sp.*), sumac (*Rhus sp.*), and gooseberries (*Ribes sp.)*. Other native fruiting plant species, like riverbank grapevine (*Vitis riparia*), raspberry (*Rubus idaeus*), and butternut tree (*Juglans cinerea*), were found. The main introduced fruiting shrubs and vines were common buckthorn (*Rhamnus cathartica*), glossy buckthorn (*Rhamnus frangula*), and Virginia creeper (*Parthenocissus quinquefolia*). The MBO is a small site (22 hectares), where the bird release locations (i.e., the banders’ cabin) is representative of the bird’s foraging habitat throughout the study site and the netting area.

The MBO and its surroundings consists of wetlands, secondary forest and shrublands at the forest-agriculture border which are valuable areas for moult-migrants arriving from the boreal forest^6,28^. Swainson’s Thrushes and Tennessee Warblers are common fall moult-migrants at the MBO, and thus ideal focal species for this research. Regarding the Tennessee Warblers’ typical migratory window at the MBO, previous studies have shown that moult migrants arrive in early August while post-moult migrants arrive more than a month later, in September^13^. Moult-migrants spend around 46 days at their stopover site compared to post-moult migrants which only occupy the site an average of 8 days^13^. This stopover duration is very similar to Swainson’s Thrushes that stay for an average of 47 (moult migrant) versus 7 (post-moult migrants) days at the same stopover site^5^.

### Sample collection and determination of fecal sample contents

From August 1 to October 15 2021, we captured after-hatch year Swainson’s Thrushes (*N* = 83) and Tennessee Warblers (*N* = 21) using 30 mm mist nets (full moult record sample population, hereafter thrushes and warblers). Each of those species were separated into two categories: moult-migrants (i.e., individuals currently moulting, *n* = 39 for thrushes and *n* = 14 for warblers), and post-moult migrants (i.e., birds that already completed their moult prior to arriving to the site and were actively migrating, *n* = 44 thrushes and *n* = 7 warblers). Several species of adult warblers and thrushes undergo a definitive prebasic moult of flight feathers at these stopover sites during moult migration^6,29^. We did not compare immature birds as they do not undergo a full (definitive) prebasic moult. We assume that birds arrived on site at the start of their moult or at least at a not very advanced moulting stage (moulting first primaries). If the bird was moulting, each flight feathers (primaries, secondaries and tertials) was scored according to a method adapted from Newton in 1966^30^. Each feather was scored as old (0), absent (0.1) or as a percentage of growth in 0.1 increments from 0.2 to 1; one meaning fully grown^2^. The captured birds were all banded, aged, and weighed. A subsample of the birds caught were sexed; 56 Swainson’s Thrushes (32 females and 20 males) and 18 Tennessee warblers (8 females and 10 males). Given the lack of sexual dimorphism in both species, we sexed the birds by obtaining 100ul blood samples and performing DNA sexing following methods in Griffiths et al*.* (^31^1998). All experimental protocols were approved under animal use protocol 2007–5446 from McGill University, and federal banding permits 10743AE and 10743 T issued by the Canadian Wildlife Service. Furthermore, all methods are reported in accordance with ARRIVE guidelines.

We were able to assess the diets on a subsample of Swainson’s Thrushes (*n* = 38) and Tennessee Warblers (*n* = 10) using fecal samples collected from the capture of each individual at the MBO (dietary subsample population). To do so, the birds were left for 10–30 min in a brown paper bag before their release. Fecal samples were collected from the bags and put in 15 ml tubes with ethanol. Samples were stored at − 20 °C until analysis. The biological samples were also collected under animal use protocol 2007–5446 and approved by the McGill University Animal Care Committee. We then sent the samples to the Canadian Center for DNA Barcoding (CCDB). They analyzed the fecal samples for arthropods and plants detection using DNA barcoding as a method of species identification^32^. A total of 54 fecal sample was sent to the CCDB laboratory (42 thrushes, with 2 recaps (*n* = 40) and 12 warblers with 1 recap (*n* = 11). There were no detections recovered by the CCBD in the diet of 2 thrushes and 1 warbler, resulting in our dietary subsample population of 48 birds (38 Swainson’s Thrushes and 10 Tennessee Warblers).

Laboratory scientists at the CCDB amplified the DNA from the fecal samples with arthropod-specific primers ZBJ-ArtF1c_t1/ZBJ-ArtR2_t1 and plant-specific primers ITS-S2F_t1/ITS4_t1^32^. Each DNA extract was amplified twice to achieve two independent PCR and sequencing replicates. The resulting sequence reads were filtered to remove low quality reads, trimmed to remove primer and adapter sequences, and then filtered for a minimum size of 100 bp. The processed reads were then compared to a comprehensive Barcode of Life Data (BOLD) reference library^33^ and assigned an identity using the Basic Local Alignment Search Tool (BLAST) algorithm^32^. The results from BLAST searches were sorted into unique taxonomic identification per sample. See Supplementary Materials for the full DNA Testing Laboratory NGS Report with detailed methods by the CCDB. Furthermore, no plant specimens were collected for this study. We assume the plant collection and use, to create the BOLD reference library, was in accordance with all the relevant guidelines. It is important to note that while this novel technology allows scientists to track the presence of food items in an individual’s diet, there are potential shortcomings to this tool to quantify proportions of food use, and to differentiate between direct and indirect consumption when multiple trophic levels are pooled together. Metabarcoding of fecal material can also yield simple barcoding errors due to DNA degradation or deficiencies in reference libraries.

### Classification of probable and improbable plant and arthropod entries

We classified if the detected arthropods and plant species found in the fecal samples of the birds were probable (i.e. present in southern Quebec) or improbable (i.e. absent from southern Quebec). Plants were assessed to species level and arthropods to the genus level, due to uncertainty about the distribution of many arthropods at the species level. We kept all detections that were deemed probable to be found in the bird’s diet at the study site. At times, some data entries provided by the lab were not as taxonomically precise as the others; some plant DNA was only identifiable at the genus-level rather than the species-level, and some arthropod entries were only identifiable at the family-rather than the genus-level. In this case, when the higher taxon was deemed probable, we accepted this entry. This resulted in keeping 53.3% of the known plant detections (32 probable plant species, which included 2 detections stopping at the genus-level). Regarding the arthropods, we deemed probable 74.3% of the arthropod detections (113 probable plant genera, which included 12 detections stopping at the family-level). To distinguish the probable versus improbable entries for the plants, we used the Canadensys Database of Vascular Plants of Canada (VASCAN), which is a comprehensive list of all vascular plants reported in Canada^34^. We also noted if the probable plants were native or introduced^34^. For the arthropods, we went through peer-reviewed articles from the journal of Zoology ‘ZooKeys’, which reported the known families of arthropods in Canada^35^. We also used iNaturalist to look at probable entries for plants and arthropods, only considering entries of “research-grade level”^36^. We eliminated species with distributions which did not overlap with the southern parts of Quebec. A table listing the probable and improbable entries of arthropods and plants can be found in the Supplementary Materials as Table S1 and S2.

### Statistical analysis of fecal contents

We used generalized linear mixed models (GLMMs) to describe how moult status, species, and seasonality might influence diet, using the ‘glmm’ function in the R package ‘rms’. For the diet, we separated the two main categories of ‘Plants’ and ‘Arthropods’ in 10 key groups to assess our hypotheses. It is important to note that the direct diet of the birds, as well as the indirect diet of the arthropods ingested by the birds or other arthropods, are amplified when conducting fecal metabarcoding. With this method, it is not currently possible to separate actual direct plant consumption versus indirect consumption via phytophagous insects. Thus, we assume plant detections are coming from a mix of directly and indirectly consumed plant material because the genetic material from some of the plants detected in the analysis is coming from the guts of consumed invertebrates. We also assume this bias is similar across our different sampling groups. Plant genetic materials were separated into either "fleshy-fruiting plant" or "dry-fruiting plant", as fleshy-fruits are more likely to be directly consumed by birds^9^ and plants without fleshy-fruit are presumably a by-product of arthropod consumption. Plant genetic materials were also separated into native versus invasive plant categories. Arthropod items were separated into their respective orders: Araneae, Coleoptera, Diptera, Hemiptera, Hymenoptera, Lepidoptera, Neuroptera, and Orthoptera. We built 12 models, each including moulting status, species and seasonality as explanatory variables and the response variable as different diet items (i.e. the four plant fruit types and eight main arthropod orders mentioned above). Interactions between predictors were not considered because none of the response variables were explained by multiple explanatory variables at the same time. Thus, since none of the individual predictors had an effect, an interaction between predictors would not either. All GLMMs were fitted with a binomial distribution since the response variable included only zeros and ones: either the diet item was detected (1) or absent (0) in the individual sample. Individual bird identification was also included as a random effect in each model to account for variation within repeated samples from the same individual. The models performed similarly with and without this random effect, which we confirmed by comparing the marginal and conditional models (see Results). Hereafter, we report statistics derived from our GLMM model. We are using an alpha level of 0.05 to assess significance and report statistical differences among groups or covariates. However, when p-values occurred between 0.1 and 0.05, we also report differences given our low sample size; such that these variables provide 'marginal' support.

Additionally, we carried out two permutational multivariate analysis of variance (PERMANOVAs) using the ‘adonis’ function in the R package ‘vegan’. Each PERMANOVA compared moult status, species, and sex to the presence of either plant or arthropod items in fecal samples, namely, (1) 17 plant orders and (2) 10 arthropod orders. We used orders to reduce the dimensionality (i.e. the number of diet-related variables) in the PERMANOVA analyses, since DNA identification to the species and genus level is less reliable. Before analyses, we removed samples where the subject’s sex was unknown and where there were no arthropod and plant items detected, which left us with a sample size 32 individuals with arthropod detected in their fecal sample and 22 individuals with plant DNA detected in their fecal sample. Note that in this analysis, individuals were double counted since the same bird could have a plant and an arthropod in its fecal sample. All analyses were performed using R Statistical Software (v4.2.1; R Core Team 2022).

## Results

After removing the improbable entries, arthropod detections were found in 47 out of 48 individuals (dietary subsample population). We detected 101 arthropod genera belonging to 71 families and 10 orders. Plant detections were found in 38 out of 48 individuals (dietary subsample population). Approximately 56% of the plant sequences recovered from all replicates showed no significant similarity to any of the publicly available records on BOLD and were assigned an identity of “unknown”^32^. Identified plant items represented approximately 30 species belonging to 23 genera, 18 families, and 17 orders. Note that this study does not represent the entire direct diet of moult migrating Swainson’s Thrushes and Tennessee Warblers. In essence, with the methods used here, it is challenging to definitively identify the plant or arthropod taxon that the birds are not eating; our understanding is limited to just a glimpse of their diet. When referring to 'eating' in this context, indirect consumption is considered, as the detected plants and arthropods could be by-products of the arthropods the birds are eating (e.g., caterpillars eating host plants or spiders eating prey).

### Species’ moult status results

Almost half of adult Swainson’s Thrushes (47%) captured (*n* = 39 out of 83) were undergoing flight-feather moult. Moulting was slightly more frequent among females (62.5%) (*n* = 20 out of 32) than males (45.8%) (*n* = 11 out of 24). Most adult Tennessee Warblers (67%, *n* = 14 out of 21) caught were undergoing moult and moulting was more frequent in females (87.5%) (*n* = 7 out of 8) than in males (50%) (*n* = 5 out of 10).

### Diet description of Swainson’s Thrushes

Plant genetic materials were detected in the fecal sample of 30 out of 38 thrushes (Table 1). However, approximately 56% of the plant entries were assigned an identity of “unknown”. Plant genetic materials from the *Rubus*, *Juglans*, and *Ambrosia* genera were found to be most present in the digestive tract of the birds (present in 13% of individuals for all 3 genera). Regarding the diet according to their moulting status, there were detections of plant genetic material from *Ambrosia*, *Boehmeria*, and *Frangula* genera for moulting birds and from *Rubus* as well as *Juglans* genera for post-moulting individuals.

**Table 1:** Plant diet differentiation among moulting and post-moulting individuals of Swainson’s Thrushes and Tennessee Warblers (dietary subsample population). The food categories are plant genera, and the results are the number and proportions of total individuals eating (directly or indirectly) each specific genus.

	Swainson’s Thrushes			Tennessee Warblers		
	# of individuals (*N* = 23)	# of moulting (*N* = 12)	# of post-moult (*N* = 11)	# of individuals (*N* = 4)	# of moulting (*N* = 4)	# of post- moult (*N* = 0)
**Fleshy fruit**						
*Arisaema*	1 (4%)	1 (8%)	0	0	0	0
*Crataegus*	1 (4%)	0	1 (9%)	0	0	0
*Celtis*	1 (4%)	0	1 (9%)	0	0	0
*Frangula*	2 (9%)	2 (17%)	0	0	0	0
*Malus*	1 (4%)	0	1 (9%)	0	0	0
*Prunus*	1 (4%)	1 (8%)	0	0	0	0
*Rhamnus*	2 (9%)	1 (8%)	1 (9%)	0	0	0
*Rhus*	1 (4%)	0	1 (9%)	0	0	0
*Rubus*	3 (13%)	0	3 (27%)	0	0	0
*Vaccinium*	1 (4%)	0	1 (9%)	0	0	0
**Dry fruit**						
*Allium*	1 (4%)	1 (8%)	0	0	0	0
*Ambrosia*	3 (13%)	2 (17%)	1 (9%)	0	0	0
*Betula*	1 (4%)	1 (8%)	0	0	0	0
*Boehmeria*	2 (9%)	2 (17%)	0	0	0	0
*Brassica*	1 (4%)	0	1 (9%)	0	0	0
*Chenopodium*	1 (4%)	1 (8%)	0	1 (25%)	1 (25%)	0
*Euphorbia*	1 (4%)	0	1 (9%)	0	0	0
*Fissidens*	1 (4%)	0	1 (9%)	0	0	0
*Glycine*	1 (4%)	0	1 (9%)	0	0	0
*Impatiens*	0	0	0	1 (25%)	1 (25%)	0
*Juglans*	3 (13%)	1 (8%)	2 (18%)	0	0	0
*Lysimachia*	1 (4%)	1 (8%)	0	0	0	0
*Poa*	1 (4%)	0	1 (9%)	0	0	0
*Rumex*	1 (4%)	1 (8%)	0	0	0	0
*Sporobolus*	1 (4%)	1 (8%)	0	0	0	0
*Solidago*	0	0	0	3 (75%)	3 (75%)	0
*Taraxacum*	1 (4%)	0	1 (9%)	0	0	0
*Trifolium*	1 (4%)	1 (8%)	0	0	0	0
*Vicia*	1 (4%)	0	1 (9%)	1 (25%)	1 (25%)	0
**Unknown**						
Total Sample size*	*N*= 30	*N* = 14	*N* = 16	*N* = 8	*N* = 7	*N* = 1
*Unknown plant material*	22 (73%)	10 (71%)	12 (75%)	6 (75%)	5 (71%)	1 (100%)

*Sample size of individuals with plant genetic material found in fecal sample (including known and unknown detections).

Arthropod items were detected in the fecal sample of 37 out of 38 thrushes (Table 2). The dominant order (present in 78.4% of individuals) was Lepidoptera. Regarding the birds’ diet according to their moulting status, species from the Lepidoptera order were the most common dietary item for both moulting and post-moulting birds. Moulting individuals also ingested Diptera, Hemiptera, Araneae, and Coleoptera, in order of frequency. Post-moulting individuals ingested Diptera, Araneae, Coleoptera, and Hemiptera.

**Table 2:** Arthropod diet differentiation among moulting and post-moulting individuals of Swainson’s Thrushes and Tennessee Warblers. The diet categories are probable arthropods orders, and the results are number and proportions of total individuals eating (directly or indirectly) each specific order.

	Swainson’s Thrushes			Tennessee Warblers		
	# of individuals (*N* = 37)	# of moulting (*N* = 17)	# of post-moult (*N* = 20)	# of individuals (*N* = 10)	# of moulting (*N* = 7)	# of post- moult (*N* = 3)
Lepidoptera (Moths and butterflies)	29 (78%)	13 (76%)	16 (80%)	5 (50%)	3 (43%)	2 (67%)
Diptera (true flies)	18 (49%)	7 (41%)	11 (55%)	5 (50%)	4 (57%)	1 (33%)
Hemiptera (true bugs)	11 (30%)	6 (35%)	5 (25%)	5 (50%)	2 (29%)	3 (100%)
Araneae (arachnids)	14 (38%)	5 (29%)	9 (45%)	5 (50%)	3 (43%)	2 (67%)
Coleoptera (beetles)	12 (32%)	4 (24%)	8 (40%)	3 (30%)	2 (29%)	1 (33%)
Orthoptera (grasshoppers, crickets)	5 (13%)	2 (12%)	3 (15%)	0	0	0
Hymenoptera (sawflies, wasps, bees, ants)	3 (8%)	1 (6%)	2 (10%)	2 (20%)	2 (29%)	0
Neuroptera (Antlions, Lacewings, and Allies)	2 (5%)	1 (6%)	1 (5%)	0	0	0
Trombidiformes (Mites)	2 (12%)	2 (12%)	1 (5%)	0	0	0
Haplotaxida (worms)	1 (3%)	0	1 (5%)	0	0	0

### Diet description of Tennessee Warblers

Plant genetic materials were detected in the fecal sample of 8 out of 10 warblers. When omitting the 56% of unknown plant entries, plant genetic materials were detected in the fecal sample of 4 out of 10 warblers (Table 1). Of those birds, 3 out of 4 had DNA of *Solidago* genus in their feces. As Solidago is not a fleshy-fruited plant, warblers may have been feeding on *Solidago* nectar or feeding on arthropods that had *Solidago* material in their digestive tracts.

Arthropod items were detected in the fecal sample of all 10 warblers captured (Table 2). Arthropod genetic materials from Lepidoptera, Diptera, Araneae, and Hemiptera orders were found to be most present in the digestive tract of the birds (present in 50% of individuals for all 4 orders). Regarding the diet according to their moulting status, species from Lepidoptera and Araneae were common dietary items for both moulting and post-moulting birds. There were more detections of Dipterans for moulting birds and Hemipterans for post-moulting birds.

### Influence of moult status, species, and seasonality on diet

**Figure 1:**
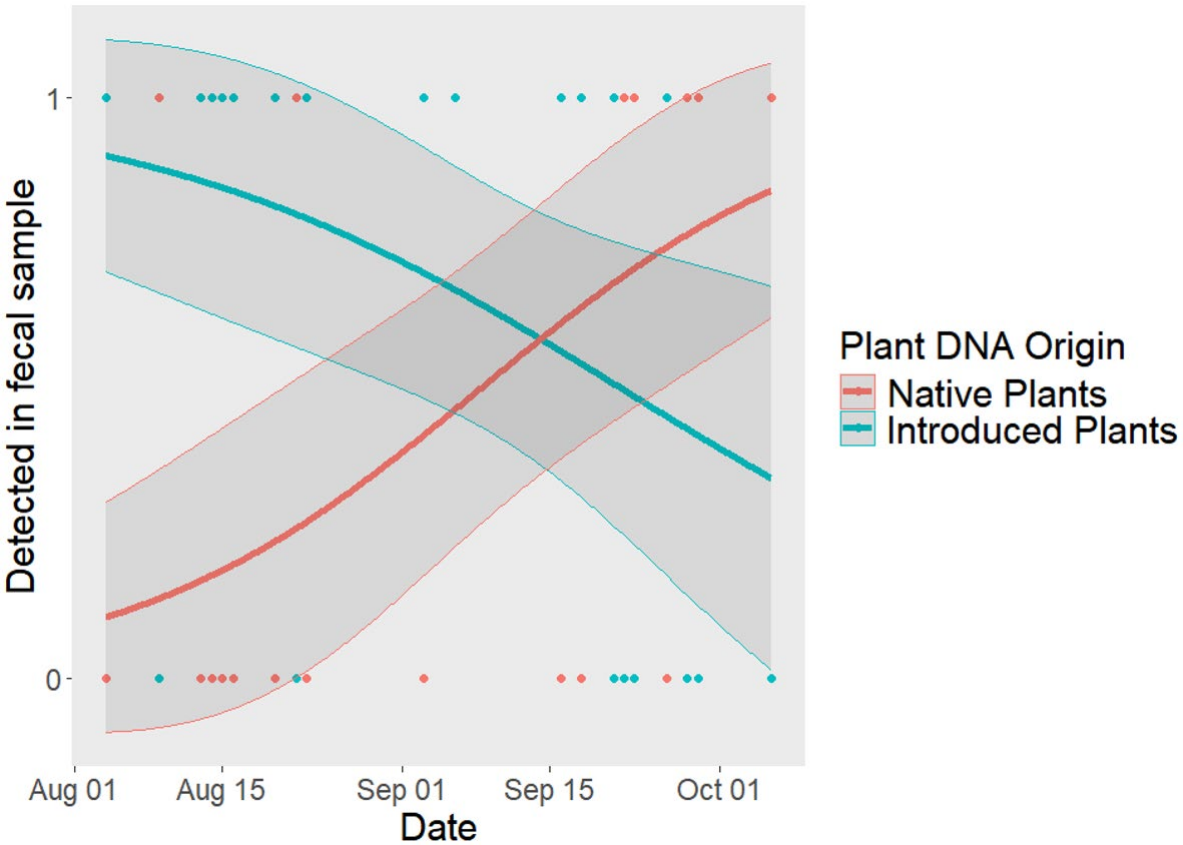
Introduced (in blue) and native (in red) plant genetic material detected (1) or not (0) in the individual’s diet according to seasonality for both Swainson’s Thrushes and Tennessee Warblers. Introduced plants tended to decrease in detection as time passed (*P* = 0.081) and native plants increased in detection as time passed (*P* = 0.021) (CI = 95%).

According to our general linearized mixed models, species and moult status were not statistically significant explanatory variables for most of the four fruit types and eight arthropod orders (Table 3). For fleshy fruit presence, however, moulting status of a bird (from both species pooled together) was found to be a significant explanatory variable (*P* = 0.04, Table 3a), such that, moulting individuals were less likely to have fleshy-fruiting plant detected in their diet than post-moult birds. More precisely, a negative trend signifies an association with post-moult migrants and a positive trend with moulting migrants. There was no variation among individuals as a comparison of the marginal and conditional models (including and excluding individual as a random effect) performed equally well or poor in every case. Finally, calendar date was positively related to the proportion of native fruit in both species’ fecal samples (*P* = 0.02) and marginally negatively related to the proportion of introduced fruit in the species’ fecal samples (*P* = 0.08, see Table 3c,d and Fig. 1). The plant species detected along with their status (native or introduced) and fruiting season can be found in Supplementary Table S3.

**Table 3:** GLMM (binomial) of whether moult status, species or date influences the detection of (a) fleshy-fruiting plant, (b) dry-fruiting plant, (c) native plant, (d) introduced plants, and (e-i) arthropod orders.

*Response variable*	*Explanatory variables*	*Estimate*	± *SE*	*Z value*	*Pr(* >*|z|)*
**(a) Fleshy fruiting plant**	**Moult status**	**-1.87**	**0.91**	**-2.06**	**0.040**
	Species	18.20	3261.32	0.01	1.00
	Date	0.03	0.02	1.39	0.16
(b) Dry-fruiting plant	Moult status	0.62	0.94	0.66	0.51
	Species	-17.59	3261.32	-0.01	1.00
	Date	-0.03	0.03	-0.98	0.33
**(c) Native plant**	Moult status	-0.69	0.84	-0.82	0.41
	Species	-1.30	1.24	-1.05	0.29
	**Date**	**0.06**	**0.03**	**2.30**	**0.021**
**(d) Introduced plant**	Moult status	-0.56	0.88	-0.64	0.52
	Species	1.95	1.25	1.55	0.12
	**Date**	**-0.05**	**0.037**	**-1.75**	**0.081**
(e) Araneae	Moult status	-0.61	0.60	-1.01	0.31
	Species	-0.50	0.72	-0.69	0.49
	Date	0.00	0.02	0.13	0.90
(f) Coleoptera	Moult status	-0.66	0.64	-1.03	0.30
	Species	0.11	0.77	0.15	0.88
	Date	0.03	0.02	1.51	0.13
(g) Diptera	Moult status	-0.25	0.58	-0.44	0.66
	Species	-0.05	0.71	-0.08	0.94
	Date	-0.00	0.02	-0.20	0.84
(h) Hemiptera	Moult status	-0.06	0.62	-0.11	0.92
	Species	-0.86	0.73	-1.18	0.24
	Date	0.00	0.02	0.21	0.83
(i) Hymenoptera	Moult status	0.41	0.96	0.42	0.67
	Species	-1.04	0.99	-1.05	0.29
	Date	-0.00	0.02	-0.13	0.89
**(j) Lepidoptera**	Moult status	-0.59	0.67	-0.88	0.38
	**Species**	**1.29**	**0.75**	**1.72**	**0.085**
	Date	0.01	0.02	0.38	0.70
(k) Neuroptera	Moult status	-0.04	1.45	-0.03	0.98
	Species	16.70	3400.72	0.01	1.00
	Date	-0.01	0.04	-0.25	0.80
(l) Orthoptera	Moult status	-0.50	0.9	-0.52	0.60
	Species	17.71	3400.72	0.01	1.00
	Date	-0.01	0.02	-0.43	0.67

Significant values are in bold. Depending on the GLMM, positive trends are either associated with higher probability of detection in (1) moult migrant rather than post-moult migrant feces, (2) Swainson's Thrushes rather than Tennessee Warbler feces, or (3) later dated rather than earlier dated feces (later being October and earlier being August).

For our PERMANOVA analyses, no difference were detected in plant or arthropod presence in the birds’ fecal samples in relation to species, moult status, or sex (See Supplementary Table S4). The only statistical difference was the interaction between species and sex on arthropod detections (*n* = 34, *p* = 0.05). See the variation in individual fecal samples in Supplementary Table S5.

## Discussion

This study effectively employed fecal DNA metabarcoding to discern species- and migration-specific dietary patterns. After excluding improbable detections, the proportion of Tennessee Warblers and Swainson’s Thrush with plant (8/10 and 30/38, respectively) and arthropod (10/10 and 37/38, respectively) detections were remarkably similar, allowing us to reject the hypothesis that warblers would have far more arthropod diet and thrushes a more plant-based diet. Instead, both species had diets composed of arthropods and fruit, however thrushes had a higher probability of consuming Lepidoptera than warblers. Post-moult migrating birds had more carbohydrate-rich foods (fleshy-fruit) than moulting birds (Table 3). Moreover, as the migration season progressed, birds reduced their consumption of introduced plant species and consumed more fruit- and energy-rich native shrubs than earlier in the season (Fig. 1).

### Diet description of study species

Our results showed that the diet of Swainson’s Thrushes was taxonomically more varied than the Tennessee Warber’s, especially for plants. Similarly, studies carried out in Illinois and Rhode Island show that 60 to 80% of Swainson’s Thrushes’ diet during their fall migration were made of wild fruits^16,24^. However, our results could also be influenced by the extremely small sample size of Tennessee Warblers with plants of known genera detected in their diet (*n* = 4), suggesting a primarily insectivorous diet. Additionally, fewer arthropod orders were found in the diet of warblers compared to thrushes. This could be explained again by the small sample size of warblers (*n* = 10) or by the fact that warblers might be more specialized and thrushes more generalist regarding arthropods in their diet. Alternatively, it could also reflect differences in arthropod diversity present in arboreal foliage compared to leaf litter. In either case, these results suggest that at our study site, the feeding niche of thrushes is broader for both plants and arthropods than the warblers’ diet.

### Importance of Lepidopteran species in diet

Our findings underscore the crucial role of Lepidopteran species in shaping the dietary habits of thrushes and warblers during their fall migration. Indeed, birds, particularly North American species, rely and prey extensively on caterpillars, since they are a high-energy, high-biomass, and nutritious food resource^37–41^ In fall, arboreal warblers and ground foragers benefit from the availability of Lepidopteran larvae, because those feed on tree leaves as well as pupate and over winter in soil and leaf litter^42^. Butler & Strazanac (2000) showed that larval composition, richness, and abundance are higher in May and August, suggesting multiple peaks in lepidopteran larvae in both spring and late summer/early fall when birds are migrating^43^. Our results are consistent with the synchronous availability and nutritious value of this food item since both thrushes and warblers showed that the highest proportion of individuals consumed species form the Lepidopteran order during their fall migration. Indeed, 78.4% of thrushes and 50% of warblers, as well as 66.7% of moulting individuals and 78.3% of post-moult individuals, had Lepidopterans detected in their diet, further suggesting that moths and butterflies are important food resource for the migrating birds in urban areas. Like mentioned previously, not being able to account for availability of prey in the area is a caveat of DNA barcoding, which means we cannot differentiate selection versus availability with certainty. However, even if Lepidoptera are occasionally detected as indirect food items, our results highlight the importance of Lepidoptera for supporting food webs by being consumed by both insectivorous birds and the predatory arthropods (e.g., spiders) that birds feed on.

Regarding our results from the fecal sample analysis, most of the plant sequences recovered from all replicates in the DNA barcoding analysis were unknown. This high proportion (56%) of unknown plant species could result from degraded DNA from being digested twice. Plant DNA may be highly fragmented by going through the gut of an herbivorous arthropod as well as the gut of an insectivorous bird, which means our inference on the importance of herbivorous arthropods to birds may be conservative. It is possible that herbivorous insects such as Lepidopteran and Symphyta larvae (Sawflies) would be the main prey insects from our unknown detections because most larval Lepidoptera are folivores^44^, highly abundant prior to pupation, and nutrient rich^45,46^. Additionally, the bird’s diet might consist of arachnids that prey on phytophagous insects. These uncertainties could be alleviated in future studies by combining methods (e.g. metabarcoding, isotopes and/or foraging data).

Our results also call attention to the importance of native plants for migrating birds as the season progresses (Fig. 1). Native plant communities support a higher diversity of caterpillar species, leading to greater bird abundance and diversity^20,47^. This relationship is supported by empirical evidence demonstrating that insect herbivores rely on plant lineages with which they have shared an evolutionary history for successful reproduction^20,47,48^. Consequently, landscapes dominated by non-native plants are less likely to support the same diversity and biomass of insect herbivores^48^, indicating that urban greenspaces with non-native plants are less supportive of breeding insectivorous birds^20^. Further studies that focus on stopover habitat quality, requirements, and communities would be beneficial since increasing native plant species in urban areas would positively impact both Lepidopteran and avian populations, contributing to the mitigation of urban biodiversity losses^47^. As well as supporting herbivorous insect communities and the birds dependent on them, prioritizing native flora in human-dominated landscapes is crucial for promoting sustainable and local food webs^20^.

### Diet community of the fall migrants

Largely, we found no statistical difference between the plant and arthropod orders present in the fecal samples of thrushes and warblers, though our small sample size may have lacked the statistical power to determine differences. Moreover, this is partly a limitation of the DNA technique which allows us to estimate detections but not quantity of prey. Not being able to identify all items eaten with the same precision with the DNA barcoding method was another limitation; some detections were unidentifiable, and others were only identifiable to genus or family level. We also could not account for availability of prey and food items in the area, or abundance of food items in diet, although we confirm what plants were available (see methods). Species and moult status had no significant effect although there was some evidence that sex (within species) impacted arthropod-related diet (*p* = 0.05). Sexual segregation of micro-foraging habitat has been observed in several songbirds while on their wintering grounds^49,50^ and breeding grounds^51–53^. Differences in foraging height, rates, and substrate contribute to these distinct niches^49,51^. Since insect families shift significantly from ground level to canopy and between plant species in temperate forests^54^, a similar distribution in foraging strategy may reflect this difference in diets between the sexes. Likewise, the difference we found in the birds’ diet based on date might also be explained by the difference in migration timing between sexes instead of habitat-usage differences *per se*^55^.

#### Arthropod community

We detected a wide distribution of arthropod diversity in the thrushes and warblers’ fecal samples. Of these, Lepidopterans and Arachnids are detected at a higher frequency in bird fecal samples and are a high-quality prey item with high caloric content^39–41^. The species in these orders also have a high quantity of crude fat, crude protein, and moisture^56^. Individuals also had lower-quality prey in their diets, such as small-bodied Hemipterans (i.e. detections of *Elasmucha, Gyponana, Scaphoideus, Lygus, Phytocoris, Hoplistoscelis, Lasiomerus*; which range in size from 6 to 10 mm (average 7.3 mm)), that contain less energy due to their small size, but are often abundant^57^. This could suggest that some diet selection for foraging birds could be based on preference and some on abundance. However, our results might be potentially recovering the diet of the predatory arthropods eaten by the bird (e.g., spiders preying on Hemipterans) and not the diet of the individual, making it difficult to assess preference.

#### Plant community

Many plant orders detected in the thrushes and warblers’ fecal samples seem to be secondary consumption products. Particularly in the warblers’ feces, many detected plants produce dry seeds that are not generally consumed by non-granivorous birds: birches (*Betula*) from the order Fagales; rapeseed (*Brassica*) from the order Brassicales; goldenrods (*Solidago*) from the order Asterales; herbaceous plants like lambsquarters and goosefoot (*Chenopodium*) from the order Caryophyllales; and grasses from the order Poales. These orders, however, are known to be host plants for a large diversity of herbivorous Lepidoptera, Symphyta, Hemiptera and Coleoptera species as well as predators and pollinators^58^. Thus, arthropod community visiting these non-fleshy fruited plants are likely an important source of arthropod food for songbirds. The Brassicales plant order consist of various trap crops and insectary plants which hosts parasitoids, as well as insects from Lepidoptera, Coleoptera, Hemiptera, and Hymenoptera^59^. Tennessee Warblers were frequently spotted foraging in fields containing those herbaceous plant species and foraging in detected tree species higher in the canopy when being tracked for an affiliated study (personal observation). Thus, we suggest that many of the plants detected in the warblers’ diet were initially eaten by arthropods, which were then eaten by the birds, since Tennessee Warblers are primarily insectivorous^25,26^. Whether the plant found in the bird’s fecal samples was directly or indirectly eaten still shows that the plant is important for the bird's diet as these plants are the foundation of local food webs. Similarly, dry plant material from various plant orders detected in the thrushes’ fecal sample are presumably a by-product of their arthropod consumption. For example, plants from the *Juglans* genus do not represent an expected dietary item for Swainson’s Thrush; it is much more likely the birds were eating herbivorous arthropods that were foraging on *Juglans* leaves. We can infer that that species from the *Juglans* genus may be important contributors to thrush diet at this stopover, even if the actual dietary items are arthropods that feed on *Juglans*. Moreover, the thrushes’ diet contained more detections of fleshy-fruiting plant genera compared to warblers, such as hackberry (*Celtis*), hawthorn (*Crataegus*), buckthorn (*Rhamnus*), Siberian crab-apple (*Malus baccata*), apple (*Malus*), bitter cherry (*Prunus emarginata*), raspberries (*Rubus*), etc. from the Rosales order as well as shrubby plants like sumac (*Rhus*) from the Sapindales order. These results are consistent with the literature, given that Swainson’s Thrushes are near-ground foragers,omnivorous, and frugivorous,especially during the fall^16,22–24^.

### Seasonality of native and non-native plant species

Seasonality influenced the detection of native and introduced plants in an individual’s diet (Fig. 1). At the start of the field season (fecal sample collection started on August 4th), there was a higher proportion of introduced plants detected in the birds’ diet (mainly buckthorn species) while, at the end of the fall migration season (last fecal sample was collected on October 6th), native plants were dominant and primarily consisted of *Rubus* species. This decrease in the detection of introduced plant species and increase of native plant species detected over time could be explained by the different fruiting times of these plant groups. Indeed, many introduced species at this site fruit in August and September: *Ambrosia artemisiifolia, Chenopodium album, Frangula alnus, Glycine max, Rubus phoenicolasius,* and *Rumex acetosa*. Some native species are found to fruit starting September: *Solidago gigantea, Solidago rugosa, Impatiens capensis,* and *Lysimachia ciliate*. Another plausible explanation could be that native plants are more adapted to the fall temperatures and are more robust, meaning that they can prosper later in the season whereas introduced plant species are not as adapted to the colder temperatures^60^. This was the case for plants on Marion Island, where invasive plants exhibited lower leaf toughness and frost tolerance than native plants^60^. This could explain why the migrating birds of our study primarily consumed the fruits of native species throughout the autumn season. Further research is needed regarding plant species invasion, fruit quality, and its impact on bird-fruit interactions.

Finally, since songbirds require abundant food resources over the length of their migratory routes, the diversity of native fruit and host plants in shrublands, forests and other green spaces, need to be maintained to provide suitable stopover sites. Unfortunately, anthropogenic alterations to the North American landscape have degraded habitat quality, introduced non-native species, and reduced the amount of greenspaces available for stopover sites used by long-distance Nearctic–Neotropical migrants^17,61^. The migration routes of many avian species follow the Atlantic or Pacific coasts where urban areas are one of the main land cover types due to the major population centers located near those coasts. Thus, migrants that encounter metropolitan areas are limited to woodlots and forests fragments within city parks as their stopover habitats^61^. Moult-migration is common in North America^7^ and moult-migrants are known to stop for long periods at these sites^5^, therefore, highlighting their diet amidst these circumstances is crucial. Further studies are needed to allow land managers in metropolitan areas to make science-based decisions regarding the survival and long-term conservation of migrating bird populations, which greatly depends on high quality stopover sites^17,61^.

## Conclusion

This paper reports novel information about the rather unknown life history period of songbird species: moult-migration. We documented the diet of Swainson’s Thrushes and Tennessee Warblers at a peri-urban stopover site during their fall migration. Many neotropical migrants spend almost as much time in large greenspaces within an urban matrix in southern Canada as they are in the boreal forest for breeding^13^, which is not currently considered thoroughly in conservation planning. Therefore, since our results suggests that diet differs according to moulting status, species and even seasonality, the management of urban areas with a variety of fruit and arthropod-rich species that can provide for the different dietary needs of the fall migrants is crucial. We advise municipality managers to plant diverse native species that can host an abundance of native species of arthropods, so that migrants can benefit from an adequate and optimal environment at their stopover site. Indeed, native landscaping positively influences songbird abundance and may reduce biodiversity losses in human-dominated landscapes^20,47^. The large-scale loss in migratory bird species have serious conservation implications that could be mitigated with changes in landscape practices, especially by the management of urban stopover sites.

### Supplementary Information

Supplementary Information 1.

## Data Availability

The data and the codes from this research have not yet been archived, but archiving can be done and shared upon request to the corresponding author.
