# Supplementary material for: Diet of moulting Swainson's Thrushes (Catharus ustulatus) and Tennessee Warblers (Leiothlypis peregrina) at a stopover site during fall migration measured with fecal DNA metabarcoding
Source: Sci Rep. 2024 Apr 30;14:9913. doi: 10.1038/s41598-024-59462-0 (PMC11061280; doi:10.1038/s41598-024-59462-0)
Supplement: Supplementary file 1 — Supplementary Information 1. [file 41598_2024_59462_MOESM1_ESM.pdf]

Title. Diet of moulting Swainson's Thrushes (*Catharus ustulatus*) and Tennessee Warblers (*Leiothlypis peregrina*) at a stopover site during fall migration measured with fecal DNA metabarcoding.

Authors. *Ana Blanc-Benigeri[1], Vanessa Poirier[1], Desiree Narango[2], Kyle H. Elliott[1], and Barbara Frei[3]

[1]Department of Natural Resources Sciences, McGill University, Montreal, Canada

[2]Vermont Center for Ecostudies, White River Junction, Hartford, VT 05001, USA

[3]Science and Technology Branch, Environment & Climate Change Canada, Montreal, Canada

***Corresponding author**. Ana Blanc-Benigeri. Montreal, QC, Canada. ana.blanc-benigeri@mail.mcgill.ca

SUPPLEMENTARY MATERIALS

Table S1. The kept (Probable) and eliminated (Improbable) detections of plants in the bird's fecal samples

Phylum	Order	Family	Genus	Probable or Improbable (Genus)	Species	Probable or Improbable (Species)
Magno-liophyta	Alismatales	Araceae	Arisaema	Probable	Arisaema triphyllum	Probable
			Lemna	Improbable	Lemna trisulca	Improbable
	Arecales	Arecaceae	Ceroxylon	Improbable	Ceroxylon weberbaueri	Improbable
	Asparagales	Alliaceae	Allium	Probable	Allium ampeloprasum	Probable
		Alliaceae	Allium	Probable	Allium sativum	Probable
	Asterales	Asteraceae	Ambrosia	Probable	Ambrosia artemisiifolia	Probable
			Solidago	Probable	Solidago gigantea	Probable
					Solidago lepida	Improbable
					Solidago rugosa	Probable
			Taraxacum	Probable	Taraxacum erythrospermum	Probable
	Brassicales	Brassicaceae	Brassica	Probable	Brassica napus	Probable
	Caryophyllales	Chenopodiaceae	Chenopodium	Probable	Chenopodium album	Probable
					Chenopodium strictum	Improbable
		Polygonaceae	Rumex	Probable	Rumex acetosa	Probable
	Ericales	Balsaminaceae	Impatiens	Probable	Impatiens capensis	Probable
					Impatiens noli-tangere	Improbable
		Primulaceae	Lysimachia	Probable	Lysimachia ciliata	Probable
					Lysimachia quadriflora	Improbable
					Lysimachia thyrsiflora	Probable
		Ericaceae	Vaccinium	Probable	Vaccinium myrtilloides	Probable
	Fabales	Fabaceae	Acacia	Improbable	NA	NA
			Aeschynomene	Improbable	Aeschynomene evenia	Improbable
			Glycine	Probable	Glycine max	Probable
			Trifolium	Probable	Trifolium pratense	Probable

			Vicia	Probable	Vicia cracca	Probable
					Vicia lathyroides	Improbable
					Vicia lutea	Improbable
	Fagales	Betulaceae	Betula	Probable	Betula alleghaniensis	Probable
					Betula lenta	Probable
		Juglandaceae	Juglans	Probable	Juglans cinerea	Probable
					Juglans nigra	Probable
	Lamiales	Acanthaceae	Justicia	Improbable	NA	NA
	Malpighiales	Calophyllaceae	Calophyllum	Improbable	NA	NA
		Euphorbiaceae	Euphorbia	Probable	Euphorbia milii	Improbable
	Poales	Poaceae	NA	Improbable	Poaceae A.guadamuz273	Improbable
			Poa	Probable	Poa pratensis	Probable
			Sporobolus	Probable	Sporobolus arabicus	Improbable
		Cyperaceae	Rhynchospora	Improbable	Rhynchospora capillacea	Improbable
	Rosales	Urticaceae	Boehmeria	Probable	Boehmeria clidemioides var. diffusa	Improbable
			Myriocarpa	Improbable	Myriocarpa longipes	Improbable
		Cannabaceae	Celtis	Probable	Celtis reticulata	Improbable
		Rhamnaceae	Frangula	Probable	Frangula alnus	Probable
					Frangula purshiana	Improbable
			Rhamnus	Probable	Rhamnus cathartica	Probable
			Sageretia	Improbable	Sageretia elegans	Improbable
		Rosaceae	Crataegus	Probable	Crataegus uniflora	Improbable
			Prunus	Probable	Prunus emarginata	Improbable
			Malus	Probable	Malus baccata	Probable
					Malus pumila	Probable
			Rubus	Probable	Rubus idaeus	Probable
					Rubus leucodermis	Improbable
					Rubus odoratus	Probable
					Rubus phoenicolasius	Probable
				Probable	NA	NA
	Sapindales	Anacardiaceae	Rhus	Probable	NA	NA
	Vitales	Vitaceae	Cissus	Improbable	NA	NA

Bryo-phyta	Fissidentales	Fissidentaceae	Fissidens	Probable	Fissidens taxifolius	Probable
	Hypnales	Hypnaceae	Hypnum	Improbable	Hypnum cupressiforme	Improbable
		Amblystegiaceae	Leptodictyum	Improbable	Leptodictyum riparium	Improbable
	Orthotrichales	Orthotrichaceae	Orthotrichum	Improbable	Orthotrichum tenellum	Improbable

Table S2. The kept (Probable) and eliminated (Improbable) detections of arthropods in the bird's fecal samples

Order	Probable or improbable (Order)	Family	Probable or improbable (Family)	Genus	Probable or improbable (Genus)
Haplotaxida	Probable	Lumbricidae	Probable	Dendrobaena	Improbable
Araneae	Probable	Agelenidae	Probable	Agelenopsis	Probable
		Amaurobiidae	Probable	Callobius	Probable
		Araneidae	Probable	Eustala	Probable
		Clubionidae	Probable	Clubiona	Probable
		Dictynidae	Probable	Emblyna	Probable
		Linyphiidae	Probable	Neriene	Probable
		Philodromidae	Probable	Philodromus	Probable
		Pisauridae	Probable	Dolomedes	Probable
		Salticidae	Probable	Eris	Probable
				Hentzia	Probable
				Pelegrina	Probable
				Tutelina	Probable
		Theridiidae	Probable	Enoplognatha	Probable
		Thomisidae	Probable	Ozyptila	Probable
Coleoptera	Probable	Brentidae	Probable	Nanophyes	Probable
		Cantharidae	Probable	Rhagonycha	Probable
		Carabidae	Probable	Harpalus	Probable
				Oxypselaphus	Probable
		Cerambycidae	Probable	Sternidius	Probable
		Chrysomelidae	Probable	Neogalerucella	Probable
				Paria	Probable
		Cleridae	Probable	Enoclerus	Probable
				Placopterus	Probable
		Coccinellidae	Probable	Propylea	Probable
		Curculionidae	Probable	Calomycterus	Probable
				Gymnetron	Improbable
				Otiorhynchus	Probable
		Scirtidae	Probable	Contacyphon	Probable
		Scraptiidae	Probable	Canifa	Probable

		Silphidae	Probable	Nicrophorus	Probable
		Staphylinidae	Probable	Dinothenarus	Probable
Diptera	Probable	Asilidae	Probable	NA	NA
		Cecidomyiidae	Probable	NA	NA
		Ceratopogonidae	Probable	Culicoides	Probable
		Ceratopogonidae	Probable	Dasyhelea	Probable
		Chironomidae	Probable	Chironomus	Probable
			Probable	Cricotopus	Probable
			Probable	Dicrotendipes	Probable
			Probable	Microtendipes	Probable
			Probable	Orthocladius	Probable
			Probable	Polypedilum	Probable
		Chloropidae	Probable	NA	NA
		Chyromyidae	Probable	NA	NA
		Culicidae	Probable	Coquillettidia	Probable
				Culex	Probable
		Drosophilidae	Probable	Drosophila	Probable
		Empididae	Probable	Empis	Probable
				Rhamphomyia	Probable
		Ephydridae	Probable	Discocerina	Improbable
		Limoniidae	Probable	Chionea	Probable
				Dicranomyia	Probable
				Limonia	Probable
				Rhipidia	Probable
		Pipunculidae	Probable	Dasydorylas	NA
				Microcephalops	NA
		Polleniidae	Probable	Pollenia	Probable
		Sarcophagidae	Probable	NA	NA
		Syrphidae	Probable	Leucopodella	Improbable
		Tabanidae	Probable	NA	NA
		Tachinidae	Probable	Strongygaster	Probable
		Tipulidae	Probable	Nephrotoma	Probable
				Tipula	Probable
Entomobryomorpha	Improbable	Entomobryidae	Improbable	Orchesella	Improbable
Hemiptera	Probable	Acanthosomatidae	Probable	Elasmucha	Probable
		Cicadellidae	Probable	Gyponana	Probable
				Scaphoideus	Probable
		Miridae	Probable	Lygus	Probable
				Phytocoris	Probable
		Nabidae	Probable	Hoplistoscelis	Probable
				Lasiomerus	Probable
Hymenoptera	Probable	Platygastridae	Probable	Leptacis	NA
		Tenthredinidae	Probable	Caliroa	Probable
				Cladius	Probable

				Dimorphopteryx	Probable
				Nematus	Probable
Lepidoptera	Probable	Bucculatricidae	Probable	Bucculatrix	Probable
		Coleophoridae	Probable	Coleophora	Probable
		Crambidae	Probable	Aglaops	NA
				Anania	Probable
				Chrysoteuchia	Probable
				Glyphodes	Improbable
		Drepanidae	Probable	Habrosyne	Probable
		Elachistidae	Probable	Blastodacna	Probable
		Erebidae	Probable	Allerastria	Improbable
				Asura	Improbable
				Baniana	Improbable
				Calidota	Improbable
				Catocala	Probable
				Dolichosomastis	Improbable
				Gorgone	Improbable
				Hypena	Probable
				Hypoprepia	Probable
				Idia	Probable
				Lymantria	Probable
				Orgyia	Probable
				Palthis	Probable
				Pyrrharctia	Probable
				Robinsonia	Improbable
				Symphlebia	Improbable
				Zanclognatha	Probable
		Gelechiidae	Probable	Chionodes	Probable
				Gelechia	Probable
				Parachronistis	Improbable
		Geometridae	Probable	Anavitrinella	Probable
				Astygisa	Improbable
				Digrammia	Probable
				Euchlaena	Probable
				Hypagyrtis	Probable
				Lytrosis	Probable
				Nephodia	Improbable
				Nychiodes	Improbable
				Opisthoxia	Improbable
				Oulobophora	Improbable
				Patalene	Improbable
				Pterocypha	Improbable
				Scopula	Probable
				Trichodezia	Probable

		Gracillariidae	Probable	Caloptilia	Probable
		Lecithoceridae	Probable	NA	NA
		Limacodidae	Probable	Cania	Improbable
		Nepticulidae	Probable	Stigmella	Probable
		Noctuidae	Probable	Belciana	Improbable
				Cropia	Improbable
				Cucullia	Probable
				Eudryas	Probable
				Euxoa	Probable
				Lacinipolia	Probable
				Mentaxya	Improbable
				noctBioLep01	NA
				Ozarba	Improbable
				Periscepta	Improbable
				Pseudorthodes	Probable
				Xestia	Probable
		Nolidae	Probable	Nola	Improbable
		Notodontidae	Probable	Lochmaeus	Probable
				Schizura	Probable
		Oecophoridae	Probable	Prepocosma	Improbable
		Phiditiidae	Improbable	Tepilia	Improbable
		Sphingidae	Probable	Sphinx	Probable
		Tortricidae	Probable	Ancylis	Probable
				Grapholita	Probable
				Thaumatotibia	Improbable
		Yponomeutidae	Probable	Swammerdamia	Probable
Neuroptera	Probable	Chrysopidae	Probable	Pseudomallada	Improbable
		Mantispidae	Probable	Mantispa	Improbable
Orthoptera	Probable	Trigonidiidae	Probable	Eunemobius	Probable
				Polionemobius	Improbable
Psocodea	Improbable	Caeciliusidae	Improbable	Valenzuela	Improbable
		Psocidae	Improbable	Blastopsocus	Improbable
				Metylophorus	Improbable
Symphypleona	Improbable	Sminthurididae	Improbable	NA	NA
Trombidiformes	Probable	Eupodidae	Probable	NA	NA

Table S3. Identification of the probable plant species detected in the bird's diet, including their status, fruiting season, and fruiting category.

Species Identification	Common Name	Status	Fruiting season	Category
Allium ampeloprasum	Wild Leek	Introduced	June-July	Dry-fruiting plant
Allium sativum	Garlic	Introduced	April-May	Dry-fruiting plant

Ambrosia artemisiifolia	Common ragweed	Introduced	July-September	Dry-fruiting plant
Arisaema triphyllum	Jack in the pulpit	Native	April-June	Fleshy-fruiting plant
Betula alleghaniensis	Yellow birch	Native	April-May	Dry-fruiting plant
Betula lenta	Sweet birch	Native	Spring	Dry-fruiting plant
Brassica napus	Canola	Introduced	May-September	Dry-fruiting plant
Chenopodium album	Lambsquarters	Introduced	July-September	Dry-fruiting plant
Frangula alnus	Alder buckthorn	Introduced	May-September	Fleshy-fruiting plant
Glycine max	Soybean	Introduced	July-September	Dry-fruiting plant
Impatiens capensis	Jewelweed	Native	June-September	Dry-fruiting plant
Juglans cinerea	Butternut	Native	May-June	Dry-fruiting plant
Juglans nigra	Walnut	Introduced	May-June	Dry-fruiting plant
Lysimachia ciliata	Fringed Loosestrife	Native	May-September	Dry-fruiting plant
Lysimachia thyrsiflora	Loosestrife	Native	May-July	Dry-fruiting plant
Malus baccata	Siberian crabapple	Introduced	April-May	Fleshy-fruiting plant
Malus pumila	Apples	Introduced	April-May	Fleshy-fruiting plant
Poa pratensis	Kentucky Bluegrass	Native	May-August	Dry-fruiting plant
Rhamnus cathartica	Common buckthorn	Introduced	May-July	Fleshy-fruiting plant
Rubus idaeus	Red raspberry	Native	May-July	Fleshy-fruiting plant
Rubus odoratus	Purple-flowered raspberry	Native	June-August	Fleshy-fruiting plant
Rubus phoenicolasius	Wine raspberry	Introduced	June-August	Fleshy-fruiting plant
Rumex acetosa	Common sheep sorrel	Introduced	June-August	Dry-fruiting plant
Solidago gigantea	Goldenrods	Native	August-October	Dry-fruiting plant
Solidago rugosa	Goldenrods	Native	August-October	Dry-fruiting plant
Taraxacum erythrospermum	Red-seeded dandelion	Introduced	June-October	Dry-fruiting plant
Trifolium pratense	Red clover	Introduced	June-September	Dry-fruiting plant
Vaccinium myrtilloides	Velvet-leaved blueberries	Native	May-June	Fleshy-fruiting plant
Vicia cracca	Cow vetch	Introduced	June-September	Dry-fruiting plant

Table S4. Results of two PERMANOVAs comparing songbird species (Tennessee Warbler and Swainson's Thrush), moult status, and sex on the detection of plant and insect items (of 18 and 13 orders, respectively) in fecal samples. Note that, under the plant PERMANOVA, there is no analysis for 'Species*Moult status' or 'Species*Moult status*Sex' because of a lack of data (i.e. there were no post-moult Tennessee Warblers with plant detected in the fecal samples).

	Df	*Sum of squares*	*Mean of squares*	*F model*	R^2	*p-value*
Insect items in fecal sample						

Species	1	0.27	0.27	1.47	0.04	0.25
Moult status	1	0.09	0.09	0.48	0.01	0.67
Sex	1	0.40	0.40	2.15	0.06	0.15
*Species*Moult status*	1	0.32	0.32	1.75	0.05	0.19
*Species*Sex*	1	0.65	0.65	3.49	0.10	**0.05**
*Moult status*Sex*	1	0.04	0.04	0.22	0.01	0.86
*Species*Moult status*Sex*	1	0.11	0.11	0.57	0.02	0.76
Residuals	26	4.82	0.19		0.72	
Plant DNA in fecal sample						
Species	1	0.64	0.64	1.57	0.07	0.14
Moult status	1	0.31	0.31	0.76	0.03	0.65
Sex	1	0.24	0.24	0.60	0.03	0.75
*Species*Sex*	1	0.49	0.49	1.19	0.05	0.36
*Moult status*Sex*	1	0.47	0.47	1.16	0.05	0.40
Residuals	18	7.38	0.41		0.77	

Table S5. Item categories detected in fecal samples of molt and post-molt Tennessee Warblers and Swainson's Thrushes captured during their fall migration at the McGill Bird Observatory in 2021 and 2022. For sex, 'M' is male and 'F' is female.

Band #	Species	Sex	Molt status	Plant	Arthropod	Introduced plant	Native plant
295032159	TEWA	M	post-molt	No	Yes	NA	NA
295032332	TEWA	F	molt	Yes	Yes	NA	NA
295032416	TEWA	F	molt	Yes	Yes	NA	NA
295032420	TEWA	F	molt	Yes	Yes	NA	NA
295032476	TEWA	M	molt	Yes	Yes	No	Yes
295032486	TEWA	F	molt	Yes	Yes	Yes	No
295032659	TEWA	F	post-molt	No	Yes	NA	NA
295032795	TEWA	M	molt	Yes	Yes	No	Yes
295032798	TEWA	M	post-molt	Yes	Yes	NA	NA
295032803	TEWA	F	molt	Yes	Yes	No	Yes
298150001	SWTH	M	post-molt	Yes	Yes	NA	NA
298150010	SWTH	M	post-molt	No	Yes	NA	NA
298150037	SWTH	M	post-molt	Yes	Yes	No	Yes
298150171	SWTH	NA	post-molt	No	Yes	NA	NA
298150225	SWTH	NA	post-molt	Yes	Yes	No	Yes
298151211	SWTH	M	molt	Yes	Yes	NA	NA
298151220	SWTH	F	molt	Yes	Yes	No	Yes
298151249	SWTH	M	post-molt	Yes	Yes	NA	NA
298151262	SWTH	F	molt	Yes	Yes	Yes	No
298151298	SWTH	F	post-molt	Yes	Yes	Yes	Yes

298151517	SWTH	NA	molt	Yes	Yes	Yes	No
298151533	SWTH	M	molt	Yes	Yes	NA	NA
298151544	SWTH	NA	molt	Yes	Yes	No	Yes
298151555	SWTH	F	molt	Yes	Yes	Yes	No
298151558	SWTH	M	molt	Yes	Yes	Yes	No
298151562	SWTH	F	molt	Yes	Yes	Yes	No
298151563	SWTH	NA	molt	No	Yes	NA	NA
298151564	SWTH	F	molt	No	Yes	NA	NA
298151567	SWTH	F	molt	Yes	Yes	Yes	No
298151570	SWTH	F	molt	Yes	Yes	NA	NA
298151573	SWTH	NA	molt	Yes	Yes	NA	NA
298151584	SWTH	F	molt	Yes	Yes	Yes	No
298151615	SWTH	M	post-molt	Yes	Yes	NA	NA
298151616	SWTH	NA	post-molt	No	Yes	NA	NA
298151718	SWTH	NA	molt	No	Yes	NA	NA
298151732	SWTH	NA	post-molt	Yes	Yes	Yes	No
298151785	SWTH	NA	post-molt	No	Yes	NA	NA
298151790	SWTH	F	post-molt	Yes	Yes	Yes	No
298151791	SWTH	NA	post-molt	No	Yes	NA	NA
298151808	SWTH	NA	post-molt	Yes	Yes	NA	NA
298151839	SWTH	F	post-molt	Yes	No	No	Yes
298151854	SWTH	NA	post-molt	Yes	Yes	Yes	No
298151876	SWTH	M	molt	Yes	Yes	No	Yes
298151890	SWTH	M	post-molt	Yes	Yes	NA	NA
298151923	SWTH	F	post-molt	Yes	Yes	Yes	Yes
298151926	SWTH	NA	post-molt	Yes	Yes	Yes	Yes
298151927	SWTH	F	post-molt	Yes	No	Yes	No
298151993	SWTH	NA	post-molt	Yes	Yes	NA	NA

Find the DNA Testing Laboratory NGS Report by the Canadian Centre for DNA Barcoding (CCDB) on the following pages.

CANADIAN CENTRE FOR DNA BARCODING

DNA Testing Laboratory NGS Report

Date of issue: July 13, 2022
Prepared by: Nguyen NguyenT.X.
Approved by: Evgeny Zakharov

CLIENT INFORMATION

Accession number: BIO-22-020
Client Name: Barbara Frei, PhD; Research Scientist
Client Address: Urban Ecology & Nature-based Climate Solutions
Environment and Climate Change Canada
351, boul. Saint-Joseph, 15th floor Gatineau K1A 0H3
Contact Name: Barbara Frei (barbara.frei@ec.gc.ca)
Vanessa Poirier (vanessa.poirier@mail.mcgill.ca)

ITEMS

Description: 56 fecal samples from birds submitted in 15ml and 5ml tubes with ethanol for arthropods (ZBJ-ArtF1c_t1 primers) and plants (ITS2 primers) detection.
Dates Received: February 10, 2022
Received From: FedEx (TRK# 2896 1236 9847)
Dates of Analysis: May 03 – May 26, 2022

METHODS

Fecal samples in 42 15mL-tubes and 14 5mL-tubes were lysed with 500 uL- 2 mL of invertebrate lysis buffer with 2 mg/mL of proteinase K (Promega). DNA was extracted from 50 uL of each homogenate. Extraction was performed using a validated glass fibre, plate-based technique employed by Ivanova, Dewaard, & Hebert (2006) (DOI: 10.1111/j.1471-8286.2006.01428.x). DNA was amplified with arthropod specific primers *ZBJ-ArtF1c_t1/ZBJ-ArtR2_t1* (DOI: 10.1111/j.1755-0998.2010.02920.x) which target a 157 base-pair (bp) fragment of the barcode region of the cytochrome *c* oxidase subunit I (COI) gene and plant-specific primers *ITS-S2F_t1/ITS4_t1* (DOI:10.1371/journal.pone.0008613), which target an approximate 350 bp fragment of the ITS2 intergenic region. Each DNA extract was amplified twice to achieve two independent PCR and sequencing replicates. PCR results were visualized using pre-cast 2% agarose E-Gels (ThermoFisher). Individual samples were tagged with IonCode universal molecular identifiers (UMIs), and all samples were pooled for sequencing. Size selection was performed on a BluePippin (Sage Science) using a 2% agarose cassette targeting sequences between 200-400 bp (*ZBJ-ArtF1c_t1/ZBJ-ArtR2_t1*) or 250-500 bp (*ITS-S2F_t1/ITS4_t1*). Sequencing was performed on an Ion Torrent S5 sequencer (200 base-read library preparation kit; 200 bp Chef protocol; 510-chip for *ZBJ-ArtF1c_t1/ZBJ-ArtR2_t1* or 400 base-read library preparation kit; 400 bp Ext Chef protocol; 520-chip for *ITS-S2F_t1/ITS4_t1*). Sequencing performance metrics are shown in Figures 1 and 2. The resulting sequence reads were associated to their source sample by the UMIs (with perfect matching), filtered to remove low quality reads (minimum quality of QV20), trimmed to remove primer and adapter sequences (reads lacking a forward primer were excluded from analysis while reads lacking a reverse primer were allowed to proceed to the next step), and then filtered for a minimum size of 100 bp. The processed reads were then compared to a comprehensive BOLD reference library (www.boldsystems.org) and assigned an identity using the BLAST algorithm. The results from BLAST searches were

aggregated into unique taxonomic identifications per sample and identifications were only accepted as genuine if they were supported by at least 100 reads that matched a reference sequence with at least 95% identity across at least 100 bp (Appendices I&II). Further molecular and bioinformatic details are available upon request. All raw data will be privately and securely stored on CCDB servers for at least three months and until storage constraints require deletions of old data. All raw data is available upon request. For methodological reference, please cite Moran *et al*, 2019.

RESULTS

Arthropod detection

As DNA from each sample was amplified two times, there were a total of 112 replicates analyzed (e.g. 1_Rep1; 1_Rep2). The S5 run resulted in over 3M raw reads (Figure 1). A total of 182,603 reads could not be assigned to a known IonCode molecular index and were excluded from all subsequent analyses. The remaining 3,488,127 reads were mapped to one of the wells with either sample DNA template or one of negative controls. The vast majority of the reads were detected in wells with sample DNA (3,264,001), while only 224,126 reads were scored from template-free wells. Arthropod detections were found in 84 replicates out of 112, or 45 out of 56 samples for replicate 1 and 39 out of 56 samples for replicate 2 (Appendix I). Detected arthropod items represented approximately 195 species belonging to 76 families and 13 orders. The most commonly detected orders were Lepidoptera, Diptera, and Araneae (Figure 3 and 4). Each detection was supported by an average of 1340 sequence reads.

Plant detection

The S5 run resulted in over 5M raw read (Figure 2). A total of 440,404 reads could not be assigned to a known IonCode molecular index and were excluded from all subsequent analyses. The remaining 4,577,170 reads were mapped to one of the wells with either sample DNA template or one of negative controls. The vast majority of the reads were detected in wells with sample DNA (4,107,793), while 469,377 reads were scored from template-free wells. Plant detections were found in 75 replicates out of 112, or 37 out of 56 samples for replicate 1 and 38 out of 56 samples for replicate 2 (Appendix II). Approximately 56% of the plant sequences recovered from all replicates showed no significant similarity to any of the publicly available records on BOLD and were thus assigned an identity of “unknown”. Detected plant items (i.e. those that could be identified) represented approximately 62 species belonging to 28 families and 18 orders. The most commonly detected orders were Rosales, Ericales, and Fagales (Figure 3 and 4). Each detection was supported by an average of 1443 sequence reads.

INTERPRETATION

While many identities are listed to species (Appendix I and II), we recommend only considering identifications reliable to the level of genus due to the short length of the molecular markers used. We do not recommend using read counts as a measure for prey abundance, but rather as a guide for the reliability of detection (i.e., the more reads upon which a detection is based, the more confident you can be in that detection). The negative controls did not produce identifiable sequence reads, suggesting that the results presented here were not compromised by laboratory-introduced contamination.

REFERENCES

Ivanova NV, deWaard JR, Hebert PDN (2006). An inexpensive, automation-friendly protocol for recovering high quality DNA. Molecular Ecology Notes, 6, 998-1002. DOI: 10.1111/j.1471-8286.2006. 01428.x

Zeale MRK, Butlin RK, Barker GLA, Lees DC, Jones G (2011). Taxon-specific PCR for DNA barcoding arthropod prey in bat faeces. Molecular Ecology Resources, 11, 236-244. DOI: 10.1111/j.1755-0998.2010. 02920.x

Chen S, Yao H, Han J, Liu C, Song J, et al. 2010 Validation of the ITS2 Region as a Novel DNA Barcode for Identifying Medicinal Plant Species. PLoS ONE 5(1): e8613. doi:10.1371/journal.pone.0008613

Moran AJ, Prosser SWJ, Moran JA. 2019. DNA metabarcoding allows non-invasive identification of arthropod prey provisioned to nestling Rufous hummingbirds (Selasphorus rufus). PeerJ 7:e6596 http://doi.org/10.7717/peerj.6596.

FIGURES

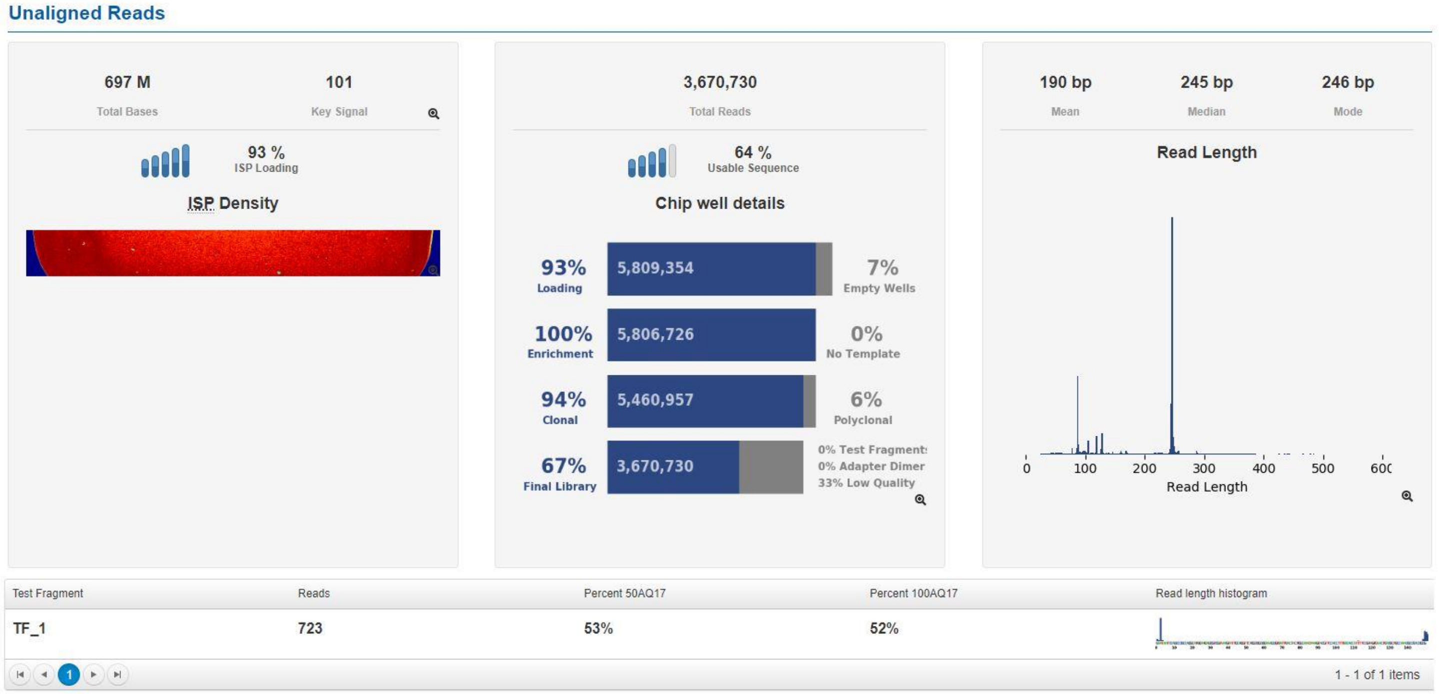

Figure 1 – Sequencing performance and test fragment metrics of the amplicon library for Arthropod detections for plate NGSFA-0135. Sequencing run name - S5-00503-479-CCDB-S5-0508 NTXN.

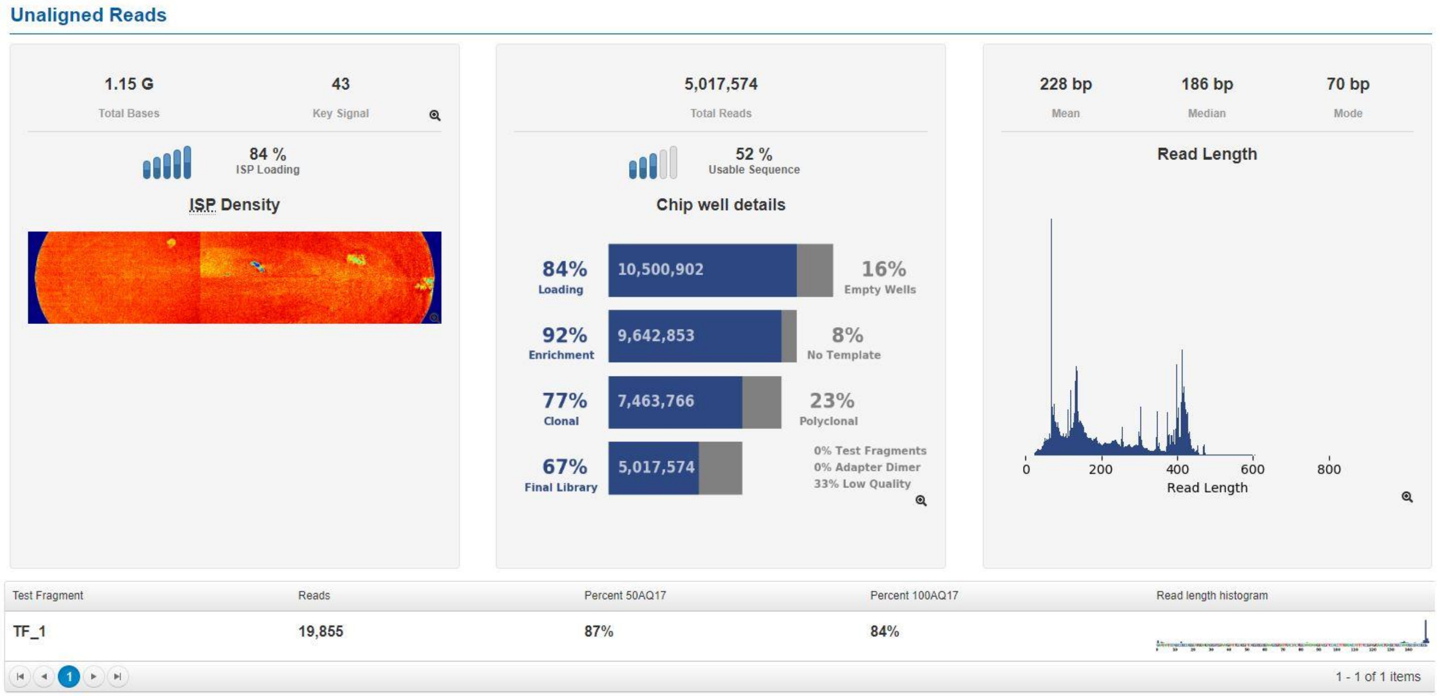

Figure 2 – Sequencing performance and test fragment metrics of the amplicon library for Plant detections for plate NGSFA-0135. Sequencing run name - S5-00503-478-CCDB-S5-0507 NTXN.

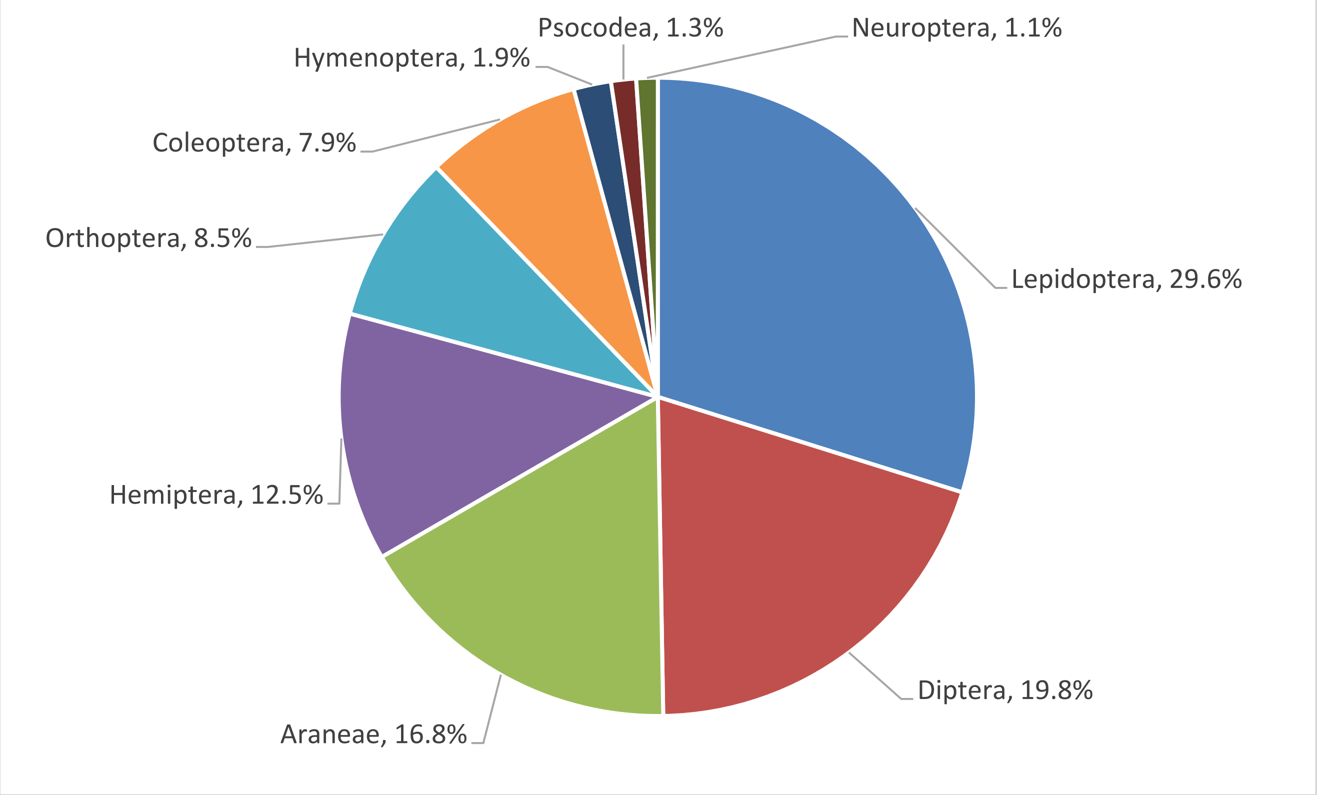

Figure 3 - Proportion of the detected Arthropod orders across the ZBJ amplifications for plate NGSFA0135 compared to a comprehensive BOLD reference library (representing 99.4% of total detections).

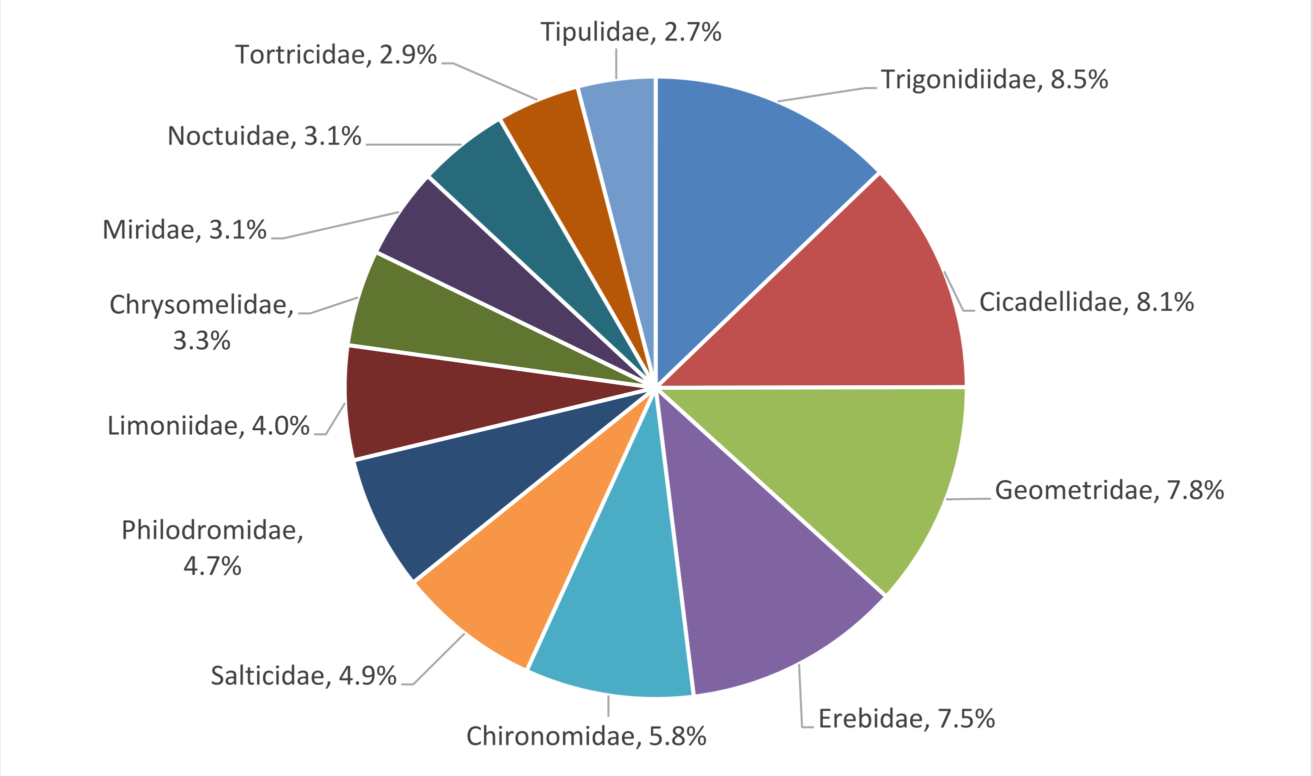

Figure 4 - Proportion of the detected Arthropod families across the ZBJ amplifications for plate NGSFA0135 compared to a comprehensive BOLD reference library (representing 66.6% of total detections)

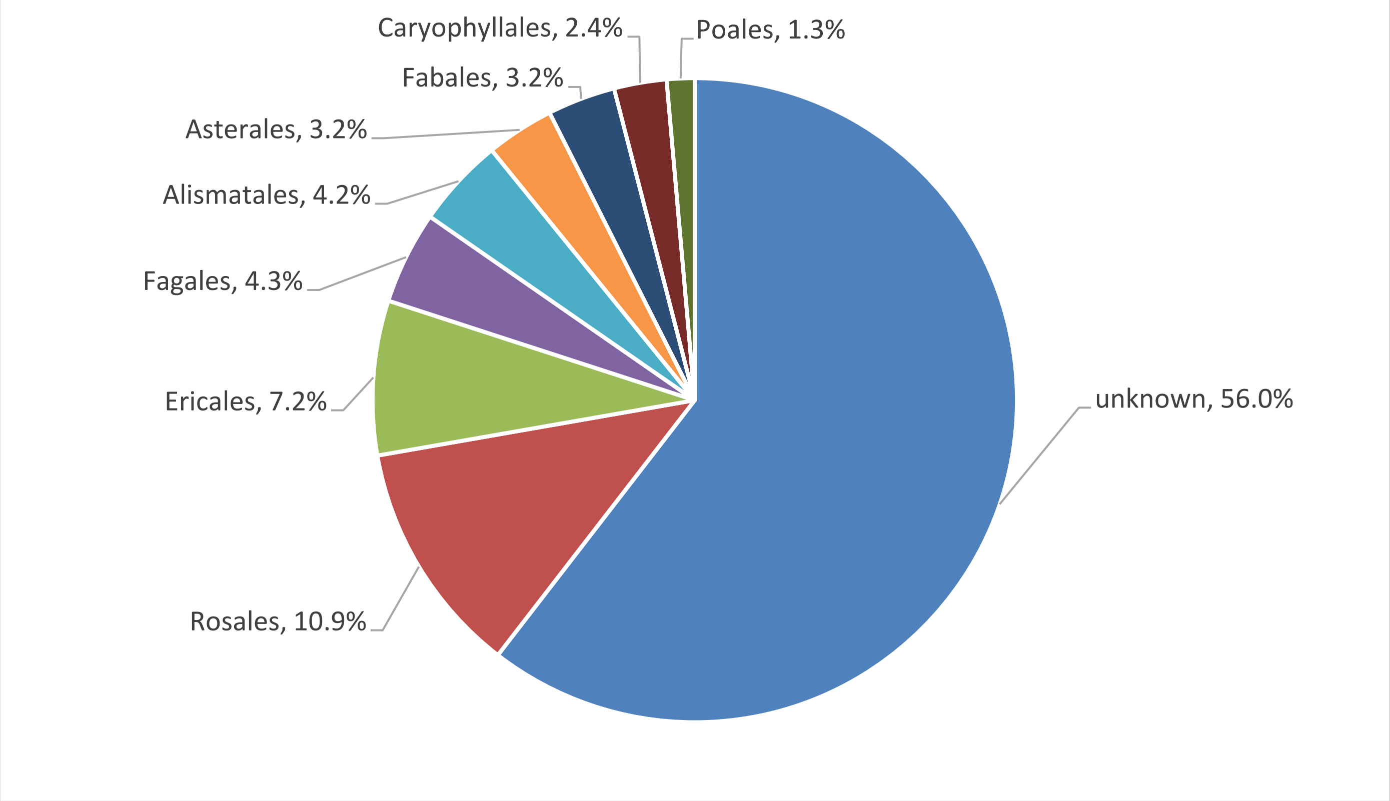

Figure 5 - Proportion of the detected plant orders across the ITS2 amplifications for plate NGSFA0135 compared to a comprehensive BOLD reference library (representing 92.5% of total detections)

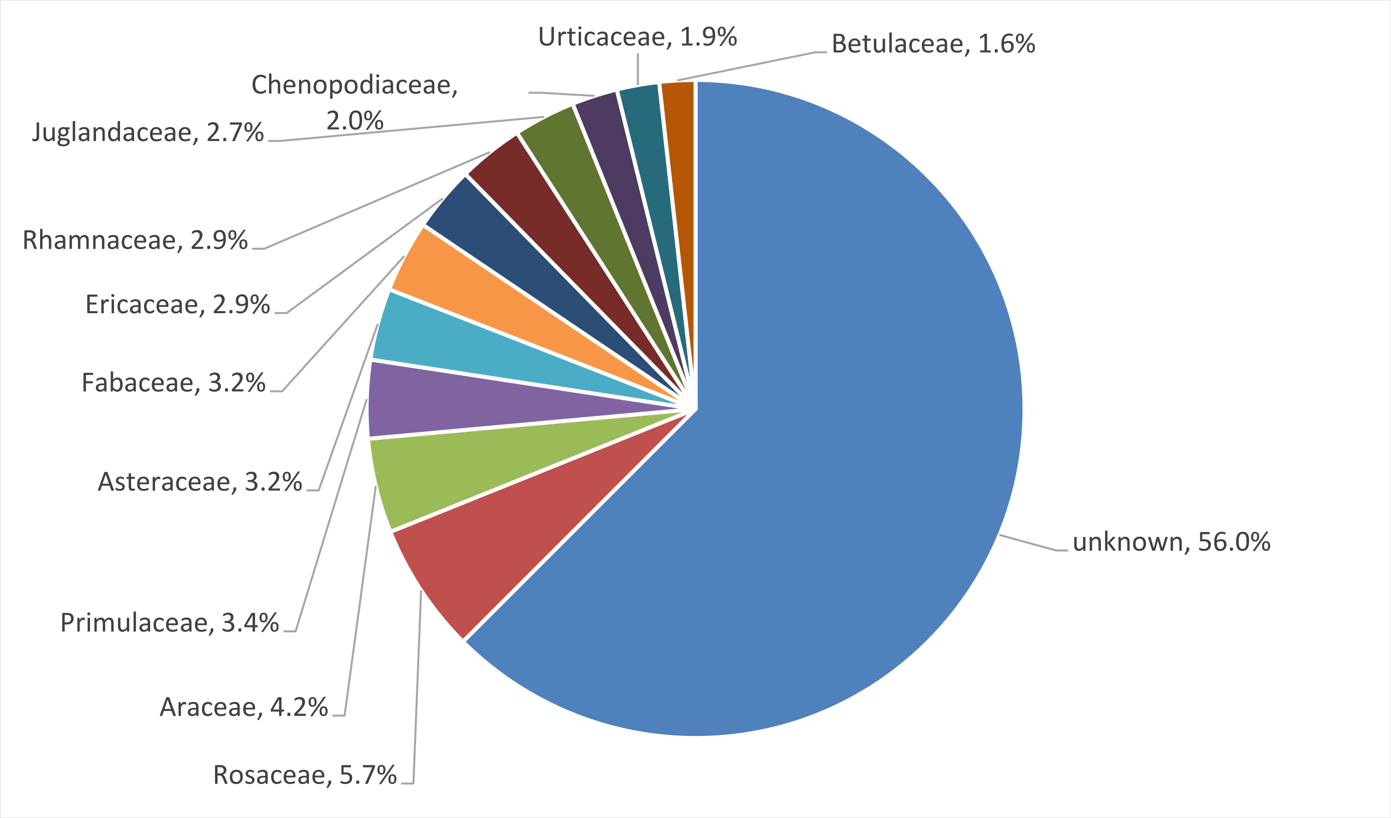

Figure 6 - Proportion of the detected plant families across the ITS2 amplifications for plate NGSFA0135 compared to a comprehensive BOLD reference library (representing 89.5% of total detections).

RESULTS REPORTED BY:

Nguyen NguyenT.X., MSc; Wildlife Forensic Technician

RESULTS REVIEWED BY:

Sean Prosser, MSc; Research Analyst

Dr. Evgeny V. Zakharov, Director, CCDB

All inquiries pertaining to this report should be directed to Nguyen NguyenT.X. (n.nguyen@uoguelph.ca) and Evgeny V. Zakharov (zakharov@uoguelph.ca).

This report should not be reproduced, except in full, without written approval of the CCDB.

Disclaimer: "THIS REPORT AND ALL ATTACHMENTS ARE CONFIDENTIAL AND SUBJECT TO SOLICITOR-CLIENT PRIVILEGE. DO NOT FORWARD, CIRCULATE, DISTRIBUTE, COPY OR DUPLICATE THIS REPORT OR ANY ATTACHMENT HERETO WITHOUT WRITTEN AUTHORIZATION"
